# Patient perspectives on the use of digital medical devices and health data for AI-driven personalised medicine in Parkinson’s Disease

**DOI:** 10.3389/fneur.2024.1453243

**Published:** 2024-12-04

**Authors:** Ivana Paccoud, Mayca Marín Valero, Laura Carrasco Marín, Noémi Bontridder, Alzhraa Ibrahim, Jüergen Winkler, Messaline Fomo, Stefano Sapienza, Fouad Khoury, Jean-Christophe Corvol, Holger Fröhlich, Jochen Klucken

**Affiliations:** ^1^Department of Precision Medicine, Luxembourg Institute of Health, Strassen, Luxembourg; ^2^Department of Digital Medicine, Luxembourg Centre for Systems Biomedicine, University of Luxembourg, Esch-sur-Alzette, Luxembourg; ^3^Asociación Parkinson Madrid (APM), Madrid, Spain; ^4^Research Centre in Information, Law and Society, Namur Digital Institute, University of Namur, Namur, Belgium; ^5^Department of Artificial Intelligence in Biomedical Engineering, Friedrich-Alexander-Universität Erlangen-Nürnberg, Erlangen, Germany; ^6^Department of Bioinformatics, Fraunhofer Institute for Algorithms and Scientific Computing, Sankt Augustin, Germany; ^7^Centre for Rare Diseases Erlangen (ZSEER), University Hospital Erlangen, Erlangen, Germany; ^8^Department of Molecular Neurology, University of Erlangen, Erlangen, Germany; ^9^Sorbonne University, Paris Brain Institute – ICM, Assistance Publique Hôpitaux de Paris, Inserm, CNRS, Pitié-Salpêtrière Hospital, Paris, France; ^10^Bonn-Aachen International Center for IT, University of Bonn, Bonn, Germany; ^11^Centre Hospitalier de Luxembourg, Luxembourg, Luxembourg

**Keywords:** Parkinson’s Disease, patient-centeredness, personalized medicine, acceptance of digital medical devices, patient preferences, use of health data, trust, Adoption of AI

## Abstract

**Introduction:**

Parkinson’s Disease (PD) affects around 8.5 million people currently with numbers expected to rise to 12 million by 2040. PD is characterized by fluctuating motor and non-motor symptoms demanding accurate monitoring. Recent advancements in digital medical devices (DMDs) like wearables and AI offer promise in addressing these needs. However, the successful implementation of DMDs in healthcare relies on patients’ willingness to adopt and engage with these digital tools.

**Methods:**

To understand patient perspectives in individuals with PD, a cross-sectional study was conducted as part of the EU-wide DIGIPD project across France, Spain, and Germany. Multidisciplinary teams including neurodegenerative clinics and patient organizations conducted surveys focusing on (i) sociodemographic information, (ii) use of DMDs (iii) acceptance of using health data (iv) preferences for the DMDs use. We used descriptive statistics to understand the use of DMDs and patient preferences and logistic regression models to identify predictors of willingness to use DMDs and to share health data through DMDs.

**Results:**

In total 333 individuals with PD participated in the study. Findings revealed a high willingness to use DMDs (90.3%) and share personal health data (97.4%,) however this differed across sociodemographic groups and was more notable among older age groups (under 65 = 17.9% vs. over 75 = 39.29%, *p* = 0.001) and those with higher education levels less willing to accept such use of data (university level = 78.6% vs. 21.43% with secondary level, *p* = 0.025). Providing instruction on the use of DMDs and receiving feedback on the results of the data collection significantly increased the willingness to use DMDs (OR = 3.57, 95% CI = 1.44–8.89) and (OR = 3.77, 95% CI = 1.01–14.12), respectively.

**Conclusion:**

The study emphasizes the importance of considering patient perspectives for the effective deployment of digital technologies, especially for older and more advanced disease-stage patients who stand to benefit the most.

## Introduction

Parkinson’s Disease (PD) is a complex neurodegenerative condition affecting approximately 8.5 million people, with the number expected to rise to 12 million by 2040 (1). The condition is characterized by a spectrum of combined motor and non-motor symptoms that fluctuate over the course of the disease, necessitating timely and accurate monitoring of treatment response, disease severity, and progression. Recent advances in Digital Medical Devices (DMDs) and related health technologies, including wearables and sensors, coupled with Artificial Intelligence (AI), hold substantial promise in addressing these requirements in both clinical and clinical research settings (2–4). By capturing precise and reliable longitudinal information regarding the daily functioning of individuals diagnosed with PD, these technologies enable accurate and objective assessments of health trajectories, aid in communication and clinical decision-making, and make it possible to evaluate treatment effectiveness (2, 5). Indeed, current assessment methods predominantly rely on clinical and patient-reported assessments, introducing numerous biases such as the experience of the clinician, patient recall, episodic assessments and inter-rater variability, which pose substantial challenges in measuring the fluctuating nature of PD symptoms (6, 7).

Despite the increasing availability and advantages of DMDs, the successful implementation of these technologies in healthcare and clinical research will depend highly on patient acceptance and engagement (8). Numerous studies in the general population have highlighted that various personal factors such as sociodemographic characteristics, digital literacy or privacy and trust concerns can hinder the use of DMDs (9–12). Regarding the acceptance of AI in healthcare, some of the major reasons behind the lack of trust are found to be the lack of responsibility attribution in terms of error, concerns over individual privacy and ‘perceived uniqueness neglect’– AI’s inability to adequately capture the unique characteristics and symptoms of individual patients (13, 14).

Studies investigating the acceptance and the use of digital tools and AI by individuals with PD generally suggest that individuals with PD are more accepting DMDs if they are younger, when they perceive their added value, and if technologies are more user-friendly. In general, they would accept DMDs if they facilitate disease management, track functionalities and symptoms, improve interactions with healthcare professionals or provide knowledge and social support (15–17). For instance, Duroseau et al. (18) studied the acceptance of DMD-based communication tools in a sample of 109 individuals with PD and found that willingness to use digital communication tools decreased with age. In addition, individuals with PD are more inclined to utilize DMDs for home care if the technology requires minimal effort, can be seamlessly integrated into their daily routine, and if they receive sufficient support from the study team (19). A study by LaBueno et al. further found that higher digital acceptance rates were associated with higher digital competencies among the users (16).

While these studies explore the factors that could influence patient acceptance of DMDs in general, there has been limited work on the perspective of individuals with PD regarding their willingness to use AI-based DMDs as well as their preferences regarding the sharing of their data for AI-driven personalized care. This lack of focus on patient needs and preferences can have major implications when implementing DMDs and AI in healthcare and clinical research, especially as it concerns complex diseases such as PD. Therefore, this study aims to investigate the determinants of the willingness to use DMDs and the collection of sensitive data for AI processing, as well as to capture patient views, concerns, and preferences related to such use while considering their sociodemographic and the clinical status.

## Materials and methods

### Study design, population and setting

This multicentre cross-sectional study was conducted across Parkinson’s patient cohorts in France, Spain, and Germany as part of the EU-wide DIGIPD project (20). The primary objective of the project was to validate the potential of digital biomarkers to support early diagnosis and personalized disease management of patients with PD. The cross-sectional survey, which is the subject of this paper, enrolled participants who had received a clinical diagnosis of Parkinson’s and provided informed consent (Review Ethical Committee Code: 22/320-E). Individuals with PD exhibiting significant cognitive impairment, intellectual disability, or other severe psychiatric conditions were excluded from participation.

### Patient recruitment

To recruit participants, a multifaceted approach was conducted, leveraging databases from collaborating organizations, national patient associations, and prominent social media platforms such as Twitter, Facebook, LinkedIn, Google+, in addition to communication channels like partner magazines. The DIGIPD project’s social networks, accessible at https://www.digipd.eu/, were also instrumental in reaching potential participants. The recruitment process involved proactive engagement by members of the DIGIPD team who sent invitations to all individuals with PD who expressed interest in being contacted for research projects. Interested participants received project information and reviewed and signed online or paper-based informed consent form. The principal investigator and a trained team member responsible for obtaining informed consent facilitated this process. Those meeting the inclusion criteria were invited to participate within the designated timeframe (January to March 2022) by e-mail or by phone.

### The development of the survey

The development of the questionnaires was informed by the literature on acceptance of digital health technologies (21, 22), as well as by the input of clinicians, researchers, individuals with PD and patient organization. The survey was divided into four main themes: (i) sociodemographic information, (ii) use of DMDs (iii) acceptance of using health data (iv) preferences for the DMDs use. The survey, initially drafted in English, was translated into French, German and Spanish using the EU survey platform’s automated translation feature. Subsequently, to ensure linguistic accuracy and cultural relevance, the translations underwent review by personnel affiliated with the project partners: the Clinical Research Centre of the Paris Brain Institute for French, the University Hospital Erlangen for German and the Association Parkinson Madrid for Spanish. This collaborative effort aimed to enhance the quality and precision of the translated survey content, aligning it with the linguistic nuances and context-specific considerations of each target language. Finally, the survey was tested for feasibility in a workshop with three PD patients and researchers. The primary objectives of the workshop were twofold: firstly, to estimate the time required for completion of the survey, and secondly to assess and ensure a comprehensive understanding of the survey content and to make necessary adjustments, ensuring the overall robustness of the survey instrument prior to its wider dissemination. The complete survey can be found in Appendix 1.

### Main study variables

#### Sociodemographic and clinical characteristics

The following variables were collected as a part of the sociodemographic characteristics: country of residence (France, Germany, Spain, Other) age categories (under 65, 65 to 75, Over 75), gender (female, male, intersex), educational level (no formal education, primary, secondary, post-secondary, bachelor degree, master degree, doctorate) added as a continuous variable in the regression model, and disease duration since diagnosis (<1 year, 1 to 5 years, 6 to 10 years, 11 to 15 years, 16 to 20 years, over 20 years) grouped across four levels due to small sample size in some categories (newly diagnosed, 1 to 5 years, 6 to 10 years and over 10 years).

#### Willingness to use DMDs, sharing health data and confidence in AI for health decision-making

Patients were asked questions about:

- their willingness to use DMDs in the healthcare context: *‘Would you use digital devices (i.e., smartphone, tablet, computer, specific wearable device – gait sensor on a shoe) if this would improve the information that your healthcare team has about you*’ with response categories (yes, no, not sure), grouped into a binary variable (“yes” or “no/not sure”).
- their acceptance of health data collection through digital tools for clinical purposes: ‘*Would you accept the use of your physical or mental state data, gathered through digital devices (i.e., smartphone, tablet, computer, specific wearable device – gait sensor on a shoe), for your medical treatment and health care purposes?’*, with response categories (yes, no and not sure), as well grouped into a binary variable (“yes” or “no/not sure”)
- their confidence in the use of AI-based clinical decision and support: ‘*Would you be confident in a healthcare decision/recommendations based on a computer calculation using formula of your data?*’, dichotomized into ‘No Confidence AI’ (I refuse such use, I am afraid of such use) and ‘Confidence in AI’ (I accept such use if it helps the physician with the diagnosis, I fully trust it).

#### Preferences and concerns

Finally, participants were asked about their preferences related to the use and functionalities of DMDs. This included preferences for particular types of DMDs (smartphones, computers with microphone and webcam, shoe sensors, headset microphone), preferred data collection settings (at home, hospital, both), and duration. Participants were also queried about their perspective on receiving feedback on the obtained measurements, instructions, and motivational messages (yes, no/not sure), and whether those functionalities would encourage their use of DMDs. Moreover, participants were asked about preferences regarding the type of instructions (animation videos, real-person videos, written manuals, pop-up messages). Lastly, participants were asked to share concerns related to the use of DMDs (abilities to handle them, privacy concerns, time-consuming, no concerns).

### Statistical analysis

In the first step, we performed a descriptive analysis of the sample characteristics and main variables concerning the use of DMDs, concerns and preferences. Next, depending on the sample size, for the categorical variables we used chi-squared or Fisher exact tests, to identify significant differences in the use and willingness to use DMDs, concerns with DMDs, trust in AI as well as preferences for data collection across various countries, sociodemographic groups and among participants with various disease durations. Post-hoc analysis was performed to analyze adjusted residuals (person residuals divided by an estimate of their standard error) (23). To ensure the clarity and meaningful interpretation of our analysis, participants categorized under ‘Other’ in the country variable were excluded from the study. We report only results where we found significant differences between study variables. Finally, we performed a logistic regression analysis to understand which clinical, sociodemographic, and support factors (such as having instructions or receiving personalized feedback) are associated with the willingness to use DMDs (Model 1) and willingness to share health data for AI (Model 2) while controlling for country effects. The predictors were estimated on an odds ratio scale, with a 95% confidence interval. The first aimed to ensure that excluding the ‘other’ category from the country analysis would not significantly alter the results. The second analysis aimed to confirm that excluding participants who responded ‘not sure’ from the analysis and grouping them with those who responded ‘no’ did not yield different results.

## Results

### Patient sociodemographic and clinical characteristics

A total of 333 individuals with PD participated in the study. France accounted for 17%, Germany 8%, Spain 64%, and the remaining 11% represented other regions. Among these participants, nearly half were below the age of 65, accounting for 49% (*n* = 162). The majority were male, making up 67% (*n* = 221), and a substantial proportion were well-educated, with 75.6% holding a university degree. Additionally, most participants (82%, *n* = 270) had been diagnosed with Parkinson’s Disease (PD) within the past 10 years. A more detailed overview of the main participant’s characteristics can be found in Table 1.

**Table 1:** Characteristics of participants (*N* = 333).

Variables	*N*	(%)
Gender		
Male	221	66.77
Female	110	33.23
Age categories (years)		
Under 65	162	48.80
65–75	108	32.53
Over 75	62	18.67
Country of residence		
France	56	16.8
Germany	27	8.11
Spain	214	64.26
Other	36	10.81
Level of education		
No primary school	10	3.00
Primary school	19	5.71
Secondary school	52	15.62
Bachelor’s degree	109	32.73
Master’s degree	103	30.93
Doctoral degree	40	12.01
PD disease duration		
<1 year	19	5.76
1–5 years	142	43.03
6–10 years	109	33.03
Over 10 years	60	18.18
Already used DMDs		
Yes	159	47.89
No	165	49.70
I’m not sure	8	2.41

DMDs, Digital Medicine Devices.

Due to non-responses in certain demographic categories (e.g., gender, age, PD duration), some categories do not reflect the full sample size. Percentages are calculated based on the number of respondents for each specific category.

### Willingness to use DMDs, share health data, and confidence in AI for clinical decision support

**Figure 1:**
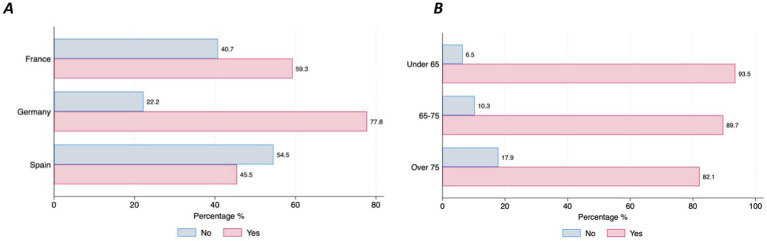
Use and Willingness to use DMDs by country and age groups. **(A)** Use of DMDs. **(B)** Willingness to use DMDs.

**Figure 2:**
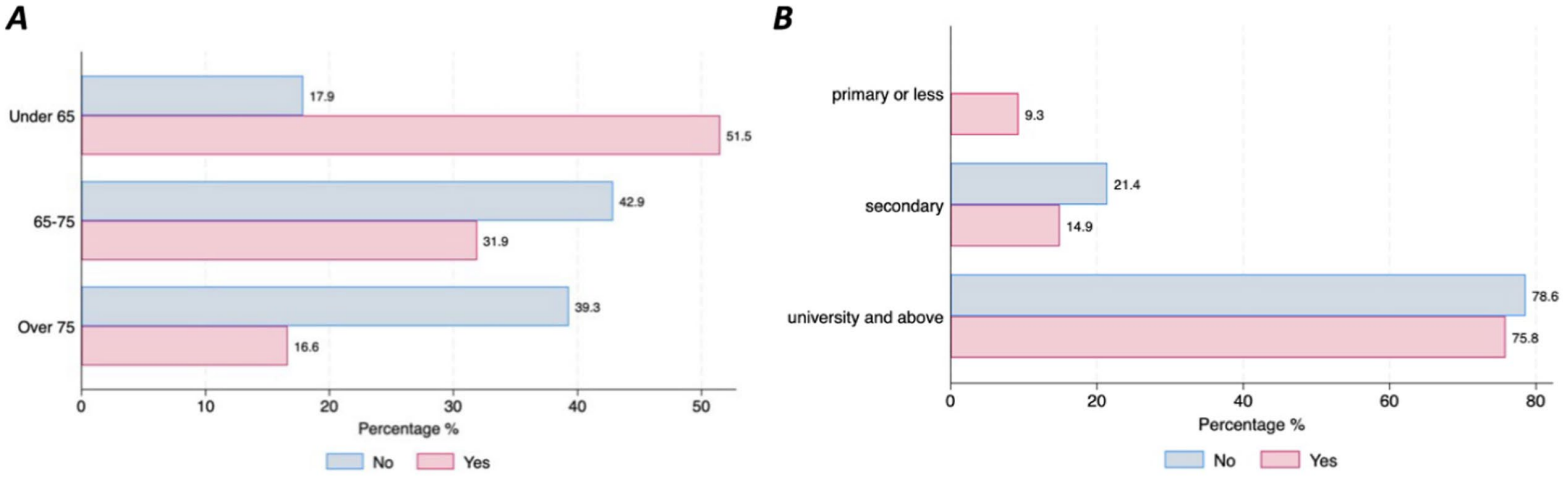
Willingness to share personal health data through DMDs for healthcare purposes by age and educational level. **(A)** Willingness to share health data by age. **(B)** Willingness to share health data by educational level.

**Figure 3:**
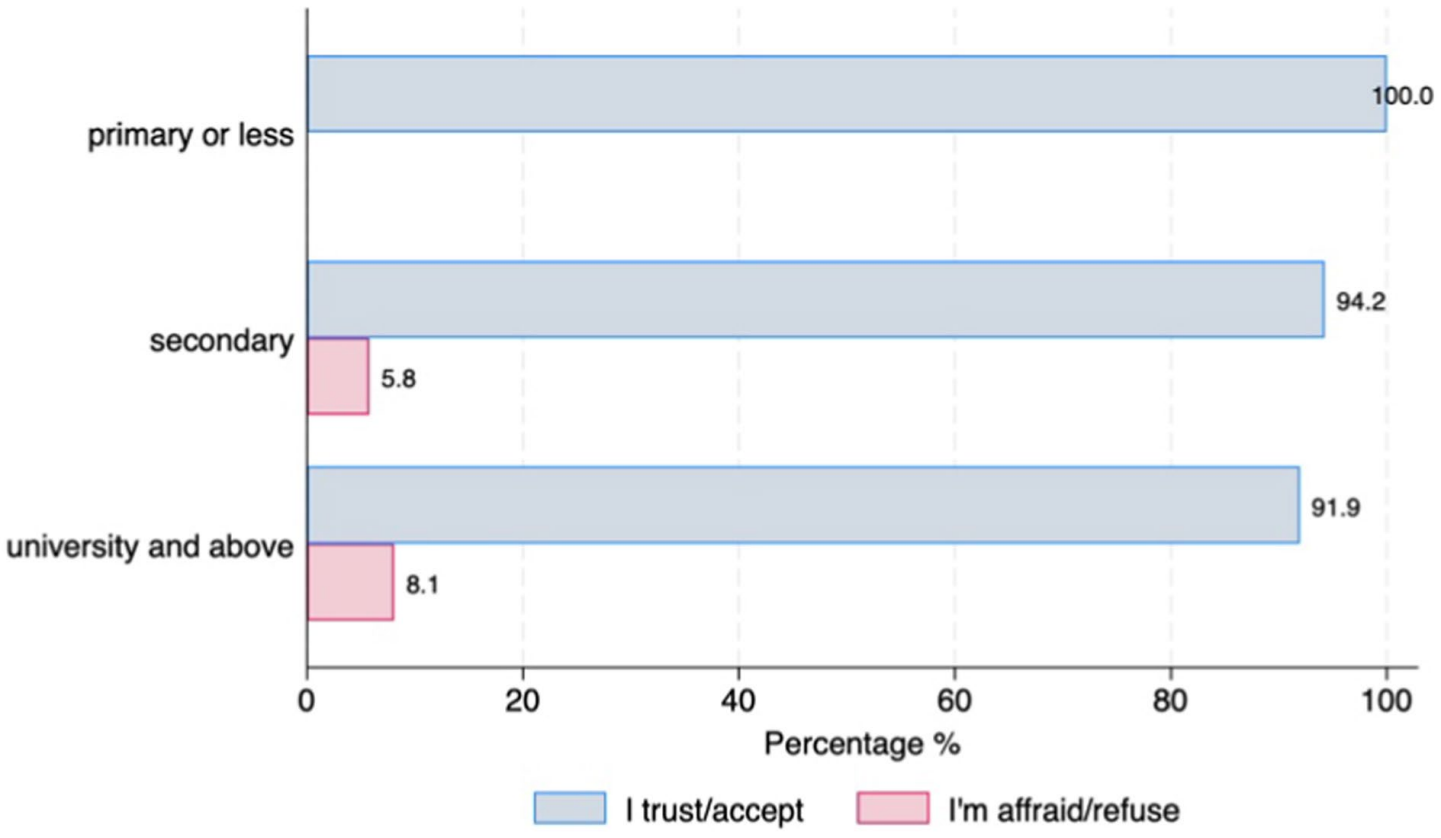
Confidence in AI for clinical decision support by educational level.

Almost half of the participants (47%; *n* = 159) have already used digital devices (i.e., smartphone, tablet, computer, specific wearable device – gait sensor on a shoe) that collect, process, and/or display personal health data, although there were differences between countries. Those living in Germany reported higher use of DMDs than those living in Spain (77.8% vs. 45.5%, *p* = 001) (Figure 1A). The majority of individuals with PD (90.3%, *n* = 278) stated that they are willing to use DMDs if that aids clinical decision-making. However, this strong commitment was lower among the older age groups (6.45% of those under 65 stating they are not willing to use DMDs vs. 17.86% of those aged over 75, *p* = 0.046) (Figure 1B). Most participants (97.4%, *n* = 302) indicated that they would accept sharing their health data collected through DMDs. However, we observed differences across age groups and educational levels, with older age groups less willing to share their health data (under 65 = 17.9% vs. over 75 = 39.29%, *p* = 0.001) (Figure 2A), and those with higher education levels less willing to accept such use of data (78.6% with university level vs. 21.43% with secondary level, *p* = 0.025) (Figure 2B). Regarding confidence in AI for clinical decision support, although most of the respondents expressed confidence in AI, those with higher educational levels (university) tended to be less likely to trust an algorithm for clinical decision support compared to those with lower educational levels (secondary or less) (8% vs. 5.8%, *p* = 0.016) (Figure 3). No other significant differences across socio-demographic groups or disease duration were observed regarding the level of confidence in AI for clinical decision support.

### Concerns related to the use of DMDs and preferences

**Figure 4:**
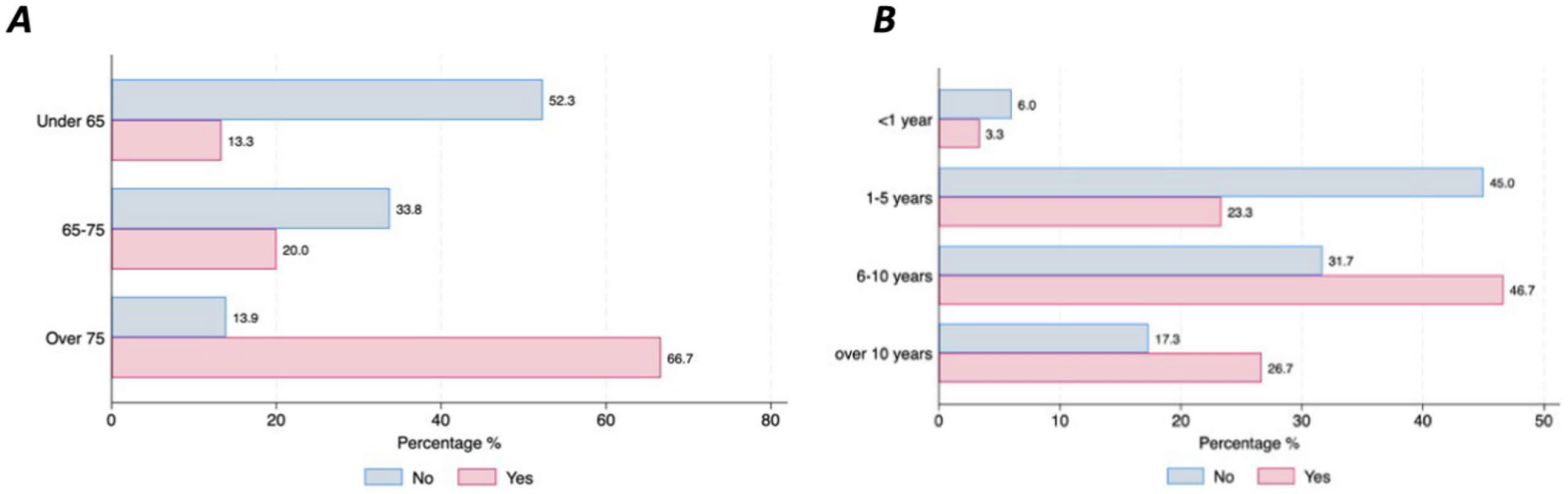
Concerns about handling DMDs by age and disease duration. **(A)** Concerns by age. **(B)** Concerns by disease duration.

Over half of the respondents with Parkinson’s Disease (63%, *n* = 210) stated that they do not have any specific concerns related to the use of DMDs. However, this differed across countries (France = 67.9%, Germany = 44.4% and Spain = 28%, *p* = 0.001). Most of the concerns about using DMDs were related to the time burden of using a device (11%) and the inability to handle the device even with support from others (9%), which was particularly salient among the older respondents (66.7% in those over 75 vs. 13.3% in those under 65, *p* = 0.000) (Figure 4A), and among those with more advanced PD duration (*p* = 0.51) (Figure 4B). Only 5% of the respondents expressed concern about sharing their health data.

**Figure 5:**
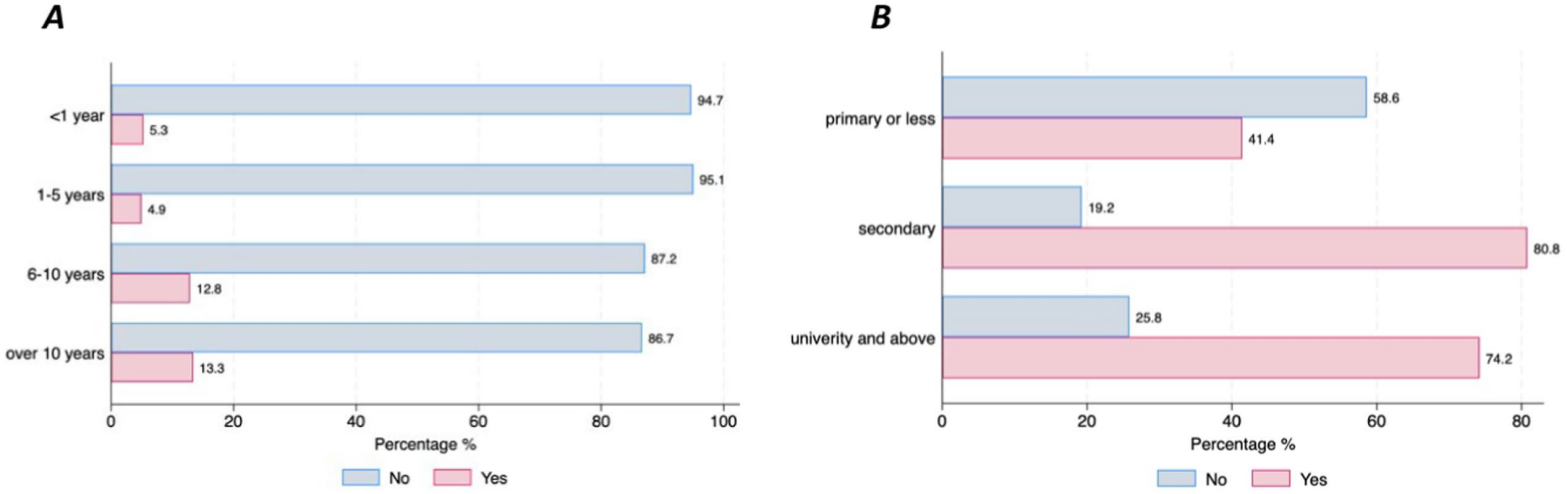
Preferences for using smartphones by disease duration and educational level. **(A)** Preferences for smartphones by PD duration. **(B)** Preferences for smartphones by education.

When it comes to the choice of DMDs, the majority of respondents (72.37%) preferred using smartphones, 20.42% preferred using a headset microphone, 30.33% expressed a preference for using a computer with a webcam and 45.05% expressed a preference for a shoe-sensor. However, differences in the level of preference for smartphones were found between those who were newly diagnosed (<1 year disease duration) and those with a longer PD disease duration (over 10 years) (94.7% vs. 61.7%, *p* = 0.016), respectively (Figure 5A), and across educational levels (primary = 41.4% and university level = 74.2%, *p* = 0.002) (Figure 5B). In terms of preferences for setting for data collection, 46% of the respondents expressed a preference for daily or monthly data collection at home, in contrast to the 3.3% who favored periodic data collection at the hospital with no statistical differences across sociodemographics or clinical status. Finally, the majority of the participants preferred to receive instructions (83%), with the most frequently preferred type of instructions being real-person videos (43.2%), followed by animation videos (38.5%). Most of the respondents also expressed preferences for feedback, such as reports on the data that has been collected (94.3%), and the use of motivational messages (68.9%). No differences in preference for instructions, feedback or motivational messages based on sociodemographic characteristics and PD duration were observed.

### Results from the logistic regression

After controlling for country effects, findings from the first logistic regression model (Model 1, Table 2), examining the relationship between willingness to use DMDs for healthcare purposes and the clinical and sociodemographic factors, and support factors, show that age as well as support factors such as having instructions and feedback are strongly associated with the willingness to use DMDs in the context of healthcare. Individuals with PD who are over the age of 75 were less likely to be willing to use DMDs in the healthcare context (OR = 0.31, 95% CI = 0.11–0.83). Having instructions (OR = 3.57, 95% CI = 1.44–8.89) and feedback, such as reports on the results of the data collection (OR = 3.77, 95% CI = 1.01–14.12), increased the willingness to use DMDs almost 4-fold, although with wide confidence intervals mostly due to the sample size.

**Table 2:** Logistic regression models presenting factors associated with the willingness to use AI-based DMDs and share health data in healthcare settings.

	Model 1			Model 2		
	Willingness to use DMD in healthcare			Willingness to share health data through DMDs for healthcare purposes		
	Odds ratio	[95% Confidence interval]		Odds ratio	[95% Confidence interval]	
Country of residence (ref: Germany)						
France	0.27	0.03	2.55	0.82	0.14	4.70
Spain	0.48	0.06	4.07	1.0	0.20	4.96
Age categories (ref: Under 65)						
65–75	0.57*	0.21	1.53	0.20***	0.06	0.63
Over 75	0.31	0.11	0.83	0.11***	0.03	0.38
Gender (ref: female)						
Male	0.93	0.39	2.23	1.06	0.43	2.61
Educational level	1.14	0.81	1.6	0.76	0.52	1.09
Disease duration (ref: newly diagnosed)						
1–5 years	0.88	0.1	7.68	1.03	0.12	9.15
6–10 years	0.72	0.08	6.41	0.71	0.08	6.43
over 10 years	0.92	0.09	9.32	1.41	0.13	15.4
Receiving instruction (ref: no)						
Yes	3.57***	1.44	8.89	3.24**	1.19	8.81
Receiving feedback (ref: no)						
Yes	3.77**	1.01	14.12	4.93**	1.37	17.72
Constant	3.1	0.09	16.25	11.793	0.45	30.17
Pseudo r-squared			0.127			0.171

***p < 0.01, **p < 0.05, *p < 0.1.

DMDs, Digital Medical Devices; ref, Reference category.

The results in the second logistic regression model (Model 2, Table 2) that investigated the association between the willingness to share health data through DMDs for healthcare purposes and sociodemographic, clinical and support factors yield similar results to the first model, show that those within the older age categories are less likely to be willing to share their health data for healthcare purposes: (OR = 0.20, 95% CI = 0.06–0.63) for those between the age of 65 and 75, and (OR = 0.11, 95% CI = 0.03–0.38) for those over the age of 75. Receiving instructions (OR = 3.24, 95% CI = 1.19–8.81) and feedback (OR = 4.93, 95% CI = 1.37–17.72) from the data collected was associated with increased odds in the willingness to share data through DMD for healthcare purposes.

## Discussion

Overall, the findings of this study demonstrate a high acceptance of DMDs and trust in AI for the purpose of personalized health, aligning with results from other studies (21, 22, 24). However, our study found that the level of use and the preferences for particular DMDs varied across participants’ country of residence as well as clinical and sociodemographic factors.

Based on a sample of individuals with PD across three large European Countries, namely France, Germany, and Spain, we found that those living in Germany were more likely to have used DMDs compared to those living in Spain, which might be due to the country’s digital readiness given the implementation of DMDs within the healthcare system. Indeed, Germany is a pioneering country that authorizes healthcare providers to prescribe Digital Health Applications so-called DIGAs (25). Furthermore, our findings indicate that individuals in the older age groups were less willing to adopt technologies. Older individuals with PD voiced higher concerns regarding their ability to manage the DMDs and concerns regarding the time burden of using the device. These results are in line with studies on acceptance of digital health technologies indicating that older adults with chronic diseases and individuals living with PD are less likely to use DMDs and more prone to express concerns related to time burden and difficulties in managing DMDs (10, 21). We also observed differences in the preferences for device technology, as individuals in more advanced disease stages and with lower education levels tended to show lower preferences toward using smartphones. This tendency might be attributed to the levels of digital literacy and challenges experienced by individuals with PD who face both motor symptoms (such as tremors, gait problems, or rigidity) and non-motor symptoms, including cognitive difficulties (26). These challenges might make handling DMDs particularly smartphones, more demanding for this subgroup. Therefore, it is imperative to offer opportunities to increase digital literacy in these populations as well as to design user-friendly DMDs that seamlessly integrate into the daily activities of individuals with PD. Previous research suggests that automating data collection through commonly used devices like watches, shoes, and jewellery could reduce the physical and mental effort of individuals with PD, consequently improving use and engagement (22, 27). In addition, our results show that the majority of the participants expressed preferences to receive instructional videos on how to use the DMDs (predominantly in the form of real-person videos), which was also shown to be strongly associated with the willingness to use DMDs in the logistic regression model. Previous studies on the acceptance of technologies confirm these findings, suggesting that having technical and social support such as instructions, and encouragement from healthcare professionals or caregivers and families are important predictors of the acceptance of digital technologies (10, 28). Furthermore, in our sample, the majority of respondents favored the concept of home monitoring over periodic monitoring and assessments in the hospital. This is expected given that most of the individuals with PD have difficulties with mobility, making it harder for them to travel to a clinic. One intriguing finding lies in the association of lower confidence in healthcare decisions based on AI and a decreased willingness to share personal health data through DMDs for healthcare purposes among those with higher levels of education. However, the relationship between education and willingness to share personal health data diminished in the regression analysis after controlling for other demographic and clinical factors, suggesting that the relationship might be confounded and factors such as age, and receiving feedback and instruction play a more important role.

Advancing efforts toward transparency regarding the use of data collected from digital technologies is a critical step in fostering trust and, consequently, increasing the willingness to share health data for AI processing. Investing in innovative approaches for privacy-perceiving digital infrastructure such as federated health records or the development of synthetic data could address privacy concerns among individuals with PD (29, 30) and allow to leverage the data to improve the health of individuals with PD. Additionally, transparency about how algorithms are developed and deployed, as well as rising awareness about the benefits of AI for clinical decision support among individuals with PD is important to increase their trust and confidence in AI. In the European Union, the General Data Protection Regulation (GDPR) overall aims to ensure lawful, fair, transparent, secure, and accountable handling of personal health information within a concise timeframe. The Regulation mandates, *inter alia*, transparent processing of personal health data, requiring clear and accessible information to be provided to patients, including purposes of processing, recipients, and data storage duration. Furthermore, patients possess legal rights to access their personal data, request rectification of their inaccuracies, obtain their erasure or processing restriction in some circumstances, and object to their processing based on individual circumstances, unless an exception applies. Importantly, patients also have the right not to be subject to a decision based solely on automated processing which significantly affects them (31).

Finally, in this article, we also show that the perceived benefits of using DMDs were strongly related to the willingness to share health data via DMDs. For instance, participants show high preferences for receiving feedback, such as reports on their health based on the collected data. The insights derived from their personalized health data can offer valuable information for individuals with PD, contributing to their higher patient engagement and empowerment. This is confirmed in previous studies, suggesting that providing feedback on the data obtained from the patients was found to be an important motivator in adherence to digital technologies (17, 32, 33), and therefore should be widely implemented.

### Limitations

Although this study foregrounds the perspectives of individuals with PD across three different European countries (France, Germany and Spain), one of the main limitations is its generalizability across all individuals with PD. In our study, the participants were mainly younger and highly educated which might overestimate the willingness to use these technologies. In addition, the majority of the participants were living in Spain, limiting the scope of country comparisons. Enhancing recruitment engagement strategies to include individuals with lower socioeconomic status necessitates collaborating with peers and community organizations, and disseminating information in simple language. Additionally, targeting locations where these communities reside can facilitate more inclusive participation. Furthermore, incorporating the experiences of healthcare professionals (34) and caregivers is essential to broaden perspectives, particularly for those in more advanced stages of the disease. While our study shows that sociodemographic and clinical characteristics of people with PD are important determinants to consider, other factors, such as the cost of DMD itself, should also be considered, especially if the DMDs are not reimbursed by the healthcare system. Lastly, it is important to note that our findings provide a general perspective rather than direct applicability to specific DMDs. Further studies assessing patient perspectives and acceptance of specific DMDs, such as smartphone apps and wrist-worn or waist-located devices, would offer valuable insights regarding the use and acceptance of specific types of technologies.

## Conclusion

Our study underscores the importance of carefully considering patients’ needs and perspectives regarding the development and deployment of DMDs for personalized care. The specific needs of older patients and patients with a more advanced disease stage need to be considered to increase adoption and meaningful engagement with DMDs as those are also the groups that could benefit the most from it. Further research should also take into account the perspective of different migrant/ethnic groups, given the structural inequalities that these groups face in the healthcare system and their specific needs and perspectives. The high enthusiasm revealed by the participants’ readiness to use digital health technology to enable better monitoring of their disease and clinical decision-making should be matched with their implementation in healthcare services. Therefore, increased patient involvement and working in partnership with researchers and clinicians is an important step toward the successful and sustainable implementation of DMDs for research and personalized healthcare. Such involvement of patients or their representatives is required by the GDPR (29).

Finally, although this was a study to understand the willingness of individuals with PD to use DMDs and share health information for the purpose of personalized care and decision support, the gap between willingness and actual use should be further explored. Indeed, although some individuals with PD are willing to use digital technologies, understanding the hurdles they face when it comes to real-time use and practical application is crucial.

## Data Availability

The raw data supporting the conclusions of this article will be made available by the authors, without undue reservation.
